# Symptoms of psychological distress amongst women during the COVID-19 pandemic in Saudi Arabia

**DOI:** 10.1371/journal.pone.0268642

**Published:** 2022-05-23

**Authors:** Ameerah M. N. Qattan

**Affiliations:** 1 Department of Health Services and Hospital Administration, Faculty of Economics and Administration, King Abdulaziz University, Jeddah, Saudi Arabia; 2 Health Economic Research Group, King Abdulaziz University, Jeddah, Saudi Arabia; University of South Carolina College of Pharmacy, UNITED STATES

## Abstract

**Background:**

Since the emergence of the COVID-19 pandemic in early 2020, several countries are still struggling to contain its spread. Apart from economic challenges, the pandemic has had a negative impact on the mental health and psychological well-being of millions of people worldwide. The effects of COVID-19 are disproportionate depending on sociodemographic characteristics. The aim of this study was to investigate the impact of COVID-19 on psychological distress among women in Saudi Arabia.

**Methods:**

Data were extracted from an online cross-sectional self-reported questionnaire conducted to measure symptoms of psychological distress during the COVID-19 pandemic in Saudi Arabia from 3 May to 8 May 2020. The study included a sample of 1527 women. The questionnaire was based on the COVID-19 Peritraumatic Distress Index (CPDI) tool to categorise women who responded to the questionnaire as experiencing normal, mild, or severe levels of distress. Sociodemographic factors related to different levels of psychological distress among women were examined using descriptive analysis and multinomial logistic regression models.

**Results:**

Overall, 44% of the respondents indicated symptoms of psychological distress due to the COVID-19 pandemic. Approximately 36% of women showed symptoms of mild psychological distress, with 8% of women experiencing a severe distress level. The results also revealed particularly high levels of psychological distress among younger women and female healthcare workers.

**Conclusion:**

The COVID-19 pandemic highly contributes to psychological distress among women in Saudi Arabia. Therefore, it is essential to establish medium- and long-term strategies that target the most vulnerable women affected by the COVID-19 pandemic.

## Introduction

Even one year after coronavirus disease 2019 (COVID-19) rocked the entire world, several countries are still struggling to contain the spread of the virus and the accompanying unprecedented death toll. The World Health Organization (WHO) declared COVID-19 a pandemic in March 2020 [1]. Unlike previous recent pandemics such as the Severe Acute Respiratory Syndrome (SARS) epidemic, the swine flu (influenza A H1N1) outbreak, Ebola, and Middle East Respiratory Syndrome (MERS), COVID-19 is a global challenge as it is a highly infectious disease [2]. The majority of affected countries have taken drastic and desperate measures to contain the virus, including social distancing, strict lockdowns, and wearing of protective equipment. COVID-19 vaccines have also been developed to assist in the fight against the pandemic [3, 4]. Despite these efforts, medical scientists continue to warn of ‘new waves’ and ‘new strains’ of the virus, which makes COVID-19 an ongoing concern.

The effects of COVID-19 on people’s lives are enormous. The strict lockdowns and social distancing have disrupted economic activities, leading to declines in businesses and employment opportunities [1]. Millions of people have lost their jobs and are confined to their homes with limited access to basic services and necessities [5]. Apart from economic challenges, COVID-19 also has a negative impact on the mental health and psychological well-being of individuals. Psychological distress, mental depression, and anxiety have been reported across the globe in countries such as Italy [6], the United States [7], Israel [8], and the Kingdom of Saudi Arabia (KSA) [9]. The uncertainty accompanying the efficacy of COVID-19 vaccines, widespread media coverage of confirmed cases and deaths, lack of sufficient necessities, and pressure of working from home are some of the drivers of increased mental and psychological issues [10].

Several studies have been conducted to uncover the psychological effects of COVID-19. Most of these studies have focused on the general public [2, 11] and healthcare workers [7, 12, 13]. Although women and children are the most vulnerable populations at times of emergencies, studies investigating their particular experience are scarce [3]. A few studies have shown that women are being disproportionately impacted by the pandemic [5, 14]. However, these studies did not exclusively focus on women, and therefore their report on the experience of women is largely limited. Consequently, there is scant empirical evidence documenting the effects of the pandemic on women. As a result, countries are prone to adopt a wholesale approach rather than a gendered approach in addressing COVID-19–related challenges [15]. This study addresses this gap in the literature by investigating the impact of COVID-19 on psychological distress specifically among women in the KSA. Employing data collected using the COVID-19 Peritraumatic Distress Index (CPDI) tool, descriptive analysis and multinomial logistic regression models were performed to identify factors contributing to symptoms of distress, which can help to provide appropriate guidance on the formulation of interventions that target vulnerable populations.

The KSA represents a compelling case to investigate the impact of COVID-19 on women for several reasons. Firstly, there exist inherent inequalities between men and women in the country. The KSA is one of the countries where women lag behind men in terms of personal income [16], putting them in a more vulnerable position owing to the generally low asset base that could cushion them against income shocks. Secondly, the measures (curfews, quarantine, and lockdowns) implemented by the Saudi government were drastic and strict, resulting in high distress for the population, including women. Furthermore, other studies have shown that women in the KSA have generally higher levels of psychological distress than men [9, 11, 17]. This context heightens the need for a study that explores and isolates the issues contributing to psychological distress during the pandemic from the perspective of women. Evidence from such research is critical for the implementation of gendered interventions in the KSA and worldwide.

## Materials and methods

### Study design and sample

This study extracted data from an online cross-sectional survey designed to assess the psychological distress experienced during the COVID-19 pandemic in the KSA, which was distributed from 3 May to 8 May 2020 [9], using a validated self-reported questionnaire [18] based on the CPDI. Participants were recruited using a simplified snowball sampling technique, where the invited participants were requested to pass on the invitations to their contacts. Data were collected online in the KSA where a link to the questionnaire was sent to the respondent through social media channels such as WhatsApp groups and Twitter. The survey was conducted in the Arabic language. The survey translated from English to Arabic by authors and was translated then back to English by different author to ensure the meaning of the content [9]. The questionnaire was divided into two main sections. The first section focused on the respondent’s sociodemographic information such as age, marital status, educational background, nationality, and work status. More information on work status was collected such as whether the respondent is a healthcare worker or otherwise. The second section of the questionnaire collected data on the self-perception on mental distress as a result of the COVID-19 pandemic.

The participants were requested to provide online informed consent before participation. The respondent needed to have been 18 years of age and a resident of the KSA to be eligible to participate in the study. The respondents were given detailed information about the aim of the study and had the freedom to withdraw at any time without explanation. They were also advised that their participation is voluntary. The participants were also assured that all information provided would be treated with the utmost confidentiality and that their opinions would remain anonymous.

According to the latest census in the KSA, the population is 34,218,169 [19]. To achieve the study objectives and have sufficient statistical power, the representative target sample size needed was calculated with a sample size calculator [20]. Using a margin of error of ±4%, a confidence level of 99%, a 50% response distribution, and 34,218,169 people, the sample size calculator arrived at 1037 participants. A total of 3036 adults successfully completed the questionnaire. Since this study restricted the analysis to female respondents, the main analysis of this study is based on a sample of 1527 women who completed the online self-administered questionnaire. A comprehensive description about the sampling method and data can be found elsewhere [9].

### Variables

Psychological distress was the dependent variable in this study. Psychological distress is defined as an uncomfortable emotion or feeling that negatively affects personal well-being, thus affecting overall functioning [21]. Psychological distress is viewed as a horrible emotion that can potentially cause a person to develop a negative feeling of the environment, other people, and self. To assess the symptoms and degree of psychological distress, respondents were asked to respond to 24 related statements on a 5-point scale as 0 = never, 1 = occasionally, 2 = sometimes, 3 = often, and 4 = most of the time to calculate the CPDI. The questions were formulated to understand almost all areas of psychological distress, including anxiety, depression, cognitive changes, specific phobias, withdrawal and compulsive behaviour, loss of social functioning, and physical symptoms.

The CPDI was constructed by summing up the scores of responses received for the 24 statements, with a range from 0 to 96. A base count of 4 was added to every response to reach the maximum standard of 100 for the CPDI. The added base facilitated comparison of the results to other studies; hence, the increase in the base had no significant effect on the gradient of the effect of COVID-19 on psychological distress symptoms. This is the same approach used in another study conducted in China [18]. To assess symptoms of psychological distress, the CPDI was classified into three levels: a normal level was indicated by a CPDI score ranging from 0 to 28, a mild distress level was obtained by a CPDI of 29 to 52, and a severe distress level was indicated by a CPDI of 53 to 100. Cronbach’s alpha statistic was used to evaluate internal reliability, which was 0.91 (P<0.001), indicating good reliability.

Sociodemographic variables, including age, marital status, education, and work status, were subdivided into different categories. Age was grouped into 18 to 29 (reference category), 30 to 39, 40 to 49, and ≥50 years. Marital status was coded as a binary variable using 0 for unmarried and 1 for married women. Education was classified as high school and below (reference category), college/university degree, and postgraduate degree. Nationality was coded as a binary variable with 0 for non-Saudi and 1 for Saudi. Work status was divided into government employment (reference category), private sector employee, self-employment, student, and unemployed.

### Statistical analysis

Data were first evaluated using descriptive statistics. The participants’ psychological distress symptoms were categorized into three distinct distress classifications according to the CPDI (normal, mild, severe), and the mean and percentage composition were calculated per distress grouping. Statistical models were corrected for multiple comparisons using the Bonferroni procedure, which divides the 0.05 P-value by the number of comparisons to reduce type 1 error [22]. This method is effective in presenting statistically significant differences across various categories with respective P-values. Moreover, multinomial regression was performed with the CPDI as the dependent variable to examine factors associated with normal, mild, and severe distress resulting from the COVID-19 pandemic. The normal distress level group was considered the reference category. The marginal effects were obtained using the first-derivative method. All analyses were conducted using STATA software (StataCorp LP, College Station, Texas, USA)

### Ethical approval

All procedures performed in this study involving human participants complied with the institutional and/or national research committee ethical standards, and with the 1964 Helsinki Declaration and subsequent amendments or equivalent ethical standards. This research has been reviewed and given a favourable opinion by King Abdulaziz University. The study was designed and conducted in accordance with the ethical principles established by King Abdulaziz University. Therefore, ethical approval was obtained from the Biomedical Ethics Research Committee, Faculty of Medicine, King Abdulaziz University (Ref-228-20).

## Results

### Sociodemographic characteristics

A total of 1527 female respondents from 13 administrative regions in the KSA participated in the study. The descriptive analysis results are presented in Table 1. Among the participants, 443 (29.01%) were healthcare workers, 597 (39.10%) were aged between 18 and 29 years, 784 (51.34%) were married, and 1437 (94.11%) were Saudi nationals. More than half of the women who participated in the study had a university or college degree.

**Table 1:** Sociodemographic characteristics of the study sample (N = 1527).

	Total	Normal	Mild	Severe	P-value
**Overall**	1527	852 (55.80)	549 (35.95)	126 (8.25)	
**Healthcare worker**					
Yes	443 (29.01)	225 (26.41)	170 (30.97)	48 (38.10)	0.059*
No	1084 (70.99)	627 (73.59)	379 (69.03)	78 (61.90)	0.059*
**Age (years)**					
18 to 29	597 (39.10)	305 (35.80)	234 (42.62)	58 (46.03)	0.182
30 to 39	525 (34.38)	291 (34.15)	185 (33.70)	49 (38.89)	0.313
40 to 49	253 (16.57)	159 (18.66)	83 (15.12)	11 (8.73)	0.010***
≥50	152 (9.95)	97(11.38)	47(8.56)	8 (6.35)	0.141
**Marital status**					
Married	784 (51.34)	439 (51.53)	281 (51.18)	64 (50.79)	0.911
Unmarried	743 (48.66)	413 (48.47)	268 (48.82)	62 (49.21)	0.911
**Education**					
High school or below	380 (24.89)	194 (22.77)	145 (26.41)	41 (32.54)	0.096*
College/University degree	890 (58.28)	503 (59.04)	317 (57.74)	70 (55.56)	0.571
Postgraduate degree	257 (16.83)	155 (18.19)	78 (15.85)	15 (11.90)	0.139
**Nationality**					
Saudi	1437 (94.11)	806 (94.60)	515 (93.81)	116 (92.06)	0.400
Non-Saudi	90 (5.89)	46 (5.40)	34 (6.19)	10 (7.94)	0.400
**Employment status**					
Government sector employee	500 (32.74)	282 (33.10)	179 (32.60)	39 (30.95)	0.699
Private sector employee	186 (12.18)	97 (11.38)	66 (12.02)	23 (18.25)	0.075*
Self-employed	46 (3.01)	28 (3.29)	17 (3.10)	1 (0.79)	0.114
Student	304 (19.91)	159 (18.66)	116 (21.13)	29 (23.02)	0.460
Unemployed	491 (32.15)	286(33.57)	171(31.15)	34 (26.98)	0.574

Data are presented as n (%)

*** P<0.01

**P<0.05

* P<0.1

The distribution of psychological distress categories according to the CPDI among the 1527 participants showed that the majority (852, 55.80%) fell in the normal range, and the remaining 675 (44.20%) participants indicated symptoms of mild or severe psychological distress. The proportion of participants (as a percentage of the total sample) who were healthcare workers increased when moving from the normal distress (26.41%) through to the mild distress (30.97%) and severe distress (38.10%) categories, which represented a significant increase at the 10% level. Although there was no significant difference in distress levels among the age groups of 18 to 29, 30 to 39, and ≥50 years, women in the age group of 40 to 49 years showed a significantly decreased trend in the proportion of participants falling in the normal distress (18.66%) through mild distress (15.12%) to severe distress (8.73%) categories, which was significant at the 1% level.

Moreover, those with an education level of high school or below showed an increasing trend from normal distress (22.77%) through mild distress (26.41%) to severe distress (32.54%), which was significant at the 10% level. There was no significant difference in the distress level trend across marital status and nationality.

### Distress level analysis

The marginal effects of multinomial logistic regression are presented in Table 2. The estimates presented represent the probability of belonging to a specific CPDI level. Healthcare workers had a higher probability of experiencing severe distress by a factor of 0.027 (P = 0.10) in comparison with non-healthcare workers. The probability of experiencing mild and severe distress decreased by 0.081 and 0.071, respectively, for those aged between 40 to 49 years relative to the other age categories. Moreover, the probability of experiencing mild and severe distress decreased with increasing education level: having a college/university degree and postgraduate degree was associated with a 0.038 and 0.067 reduction in the probability of experiencing severe symptoms of distress, respectively (P = 0.05), in comparison with having a high school level education or below. Marital status, nationality, and employment status did not have a significant impact on symptoms of psychological distress caused by the COVID-19 pandemic.

**Table 2:** The marginal effects of sociodemographic characteristics on distress level (N = 1527).

Dependent variable: CPDI	(1)	(2)	(3)
	Normal	Mild	Severe
**Healthcare worker** (No: ref)			
Yes	-0.058*	0.030	0.027*
	(0.030)	(0.029)	(0.016)
**Age** (18 to 29 years: ref)			
30 to 39 years	0.066*	-0.058*	-0.008
	(0.036)	(0.035)	(0.019)
40 to 49 years	0.152***	-0.081*	-0.071**
	(0.045)	(0.043)	(0.029)
≥50 years	0.149***	-0.099**	-0.050
	(0.051)	(0.050)	(0.032)
**Marital status** (Unmarried: ref)			
Married	-0.053*	0.031	0.022
	(0.030)	(0.028)	(0.017)
**Education level** (High school or below: ref)			
College/University degree	0.073**	-0.035	-0.038**
	(0.032)	(0.031)	(0.017)
Postgraduate degree	0.114**	-0.048	-0.067**
	(0.045)	(0.044)	(0.026)
**Nationality** (Non-Saudi: ref)			
Saudi	0.039	-0.020	-0.019
	(0.054)	(0.052)	(0.027)
**Employment status (**Government sector employee: ref)			
Private sector employee	-0.013	-0.015	0.028
	(0.044)	(0.043)	(0.022)
Self-employed	0.079	0.025	-0.103
	(0.084)	(0.078)	(0.077)
Student	0.045	-0.032	-0.012
	(0.049)	(0.047)	(0.026)
Unemployed	0.046	-0.025	-0.021
	(0.037)	(0.036)	(0.021)
**Observations**	1527	1527	1527

Standard errors are in parentheses

*** P<0.01

** P<0.05

* P<0.1

## Discussion

This study investigated the impact of COVID-19 on symptoms of psychological distress among women in the KSA. Unlike most related studies that focused on the general public and healthcare workers [7, 9, 11, 12], this study has focused exclusively on women. Women can be particularly prone to psychological distress as they occupy a lower rank in society and lack economic options [5]. Moreover, women are in a more vulnerable position to experience emotional stress compared to men and are therefore more likely to be affected mentally and psychologically by COVID-19 stressors [15]. By focusing on women, this study can provide guidance to help ensure that the COVID-19 pandemic does not further aggravate prevalent gender inequalities and vulnerabilities. Apart from descriptive analysis, this study employed multinomial logistic regression models to enhance the authenticity and reliability of the results in understanding the psychological effects of COVID-19.

The results showed that 44% of the female respondents in the KSA have been experiencing symptoms of psychological distress due to the COVID-19 pandemic. Approximately 36% of women indicated symptoms of mild psychological distress, with 8% experiencing severe distress levels. These results are consistent with findings reported in Nigeria [1], India [5], and the KSA [9, 11]. Al-Hanawi et al [9] established that the proportion of women in the KSA demonstrating normal through mild to severe distress levels exhibited an increasing trend, whereas this trend was decreasing for men. Another study found psychological distress in 35% of the respondents, with female respondents expressing more distress than their male counterparts [18]. Due to the lockdown measures, and as people have been confined to their homes, women are at potentially at a greater risk of severe distress because of pre-existing intrahousehold inequalities in division of labour [23].

The work-family spill over has an undeniable influence on health and well-being [23]. Women may experience higher distress levels due to increased childcare and unpaid household tasks [14]. This increased distress level can be attributed to the high chances of women losing their jobs and spending less time on paid work because of the pandemic [23]. Azeez et al [5] reported that migrant women in India skipped meals and compromised their sanitary and hygienic needs due to limited access to resources amidst the pandemic. In addition, women have less or no source of entertainment at home relative to men who may have smartphones and may be allowed to go outside to fetch items required for the household [5]. These findings underscore the need to understand pre-existing inequalities that may lead to negative health impacts, which must be prevented and mitigated using targeted policy interventions.

There is consistency in the results from the descriptive analysis and multinomial logistic regression regarding the symptoms of psychological distress among women who are healthcare workers, showing a higher probability of experiencing severe distress by a factor of 0.027 (P = 0.10). The proportion of participants that were healthcare workers increased when moving from the normal distress (26.41%) through to mild distress (30.97%) and severe distress (38.10%) categories, which was significant at the 10% level. This agrees with findings in the literature indicating that distress levels were high among nurses and respiratory therapists [12], workers in the medical field in the KSA [11], and frontline healthcare workers in China [10].

In studying health profession students, Li et al [13] found that 27% of the students experienced significant psychological distress due to the pandemic. This high distress level can be attributed to the direct patient care that healthcare workers provide, which places them at greater risk for infection [7]. The fear of direct contact with COVID-19 patients leads to sleep disturbances, anxiety, and depression among health professionals. Casagrande et al [6] reported that 57.1% of participants had challenges with sleep, 32.1% were anxious, and 41.8% suffered distress due to COVID-19. Therefore, there is a need to offer special support to healthcare workers to reduce the impact of the pandemic on their mental and psychological well-being.

Regarding education level, this study found that the probability of experiencing mild and severe distress symptoms decreased as the level of education increased. Having a college/university degree and postgraduate degree was related to a reduction in the probability of exhibiting symptoms of severe psychological distress by 0.038 and 0.067, respectively (P = 0.05), in comparison with the probability among those with lower levels of education. Moreover, among women with an education level of high school and below, there was an increasing trend in the proportion falling in the normal distress (22.77%) through to mild distress (26.41%) to severe distress (32.54%) categories, which was significant at the 10% level. Higher education is associated with increased income potential and is a correlate of higher quality of life [24]. Thus, a greater income potential that comes with higher levels of education would act as a cushion that mitigates the adverse psychological impact of the pandemic.

Furthermore, this study found that young women experienced more psychological distress than older women. Although there was no significant difference in the proportions of women in the age groups of 18 to 29, 30 to 39, and ≥ 50 years for different degrees of distress, those between the ages of 40 to 49 years showed a significant decreasing trend in the proportion of participants that fell in the categories of normal distress (18.66%) through mild distress (15.12%) to severe distress (8.73%). The probability of experiencing mild and severe distress decreased by a factor of 0.081 and 0.071, respectively, for those aged between 40 to 49 years. Similar results were reported in China [18], the KSA [25], and Israel [8]. The high distress levels among younger women is potentially attributable to the high use of social media, which exposes them to both information and misinformation about the virus, making them susceptible to fear, panic, and distress [1]. This necessitates the establishment of tele-mental health services to help reduce the negative effect of social media on the young population.

The results also showed an insignificant impact of marital status, nationality, and employment status on psychological distress caused by the COVID-19 pandemic; there was no significant difference in the distress trend across these categories. This result is in contrast with most of the findings in literature [10, 25, 26]. For example, Joseph et al [2] used multivariate analysis to establish high distress levels among non-Saudi nationalities compared to Saudi nationals. However, the observed patterns in these previous studies are attributable to the general population. Since the current study focused entirely on women, such trends were not observed. This is a possible indicator that being a woman dominates the experiences in a pandemic over the socio-demographic divides that exist between women. The case of women in healthcare would then be considered as an exception.

This study is unique in several ways. Firstly, it focused entirely on the psychological distress impact of COVID-19 on women, making it relevant in the design of gender-sensitive interventions that consider pre-existing gender inequalities. Secondly, the tool used for data collection, the CPDI, is reliable and validated [18]. Nevertheless, there are a few limitations of the study. This study relied on self-reported data, which has limited reliability compared to objectively assessed information. The cross-sectional design of the study also provides only a transient view of women’s psychological responses. Therefore, longitudinal studies might be needed to evaluate the stability of the responses over time. These issues ought to be considered in future studies.

## Conclusions

Employing descriptive analysis and multinomial logistic regression, with data collected using the CPDI, this study investigated the impact of COVID-19 on symptoms of psychological distress among women in the KSA. The results showed that 44% of women exhibit symptoms of psychological distress due to the COVID-19 pandemic. Approximately 36% of women suffered from mild symptoms of psychological distress, with 8% experiencing symptoms of severe distress. The results also revealed particularly high levels of psychological distress among female healthcare workers and younger women, with lower stress levels indicated among women with higher levels of education. Since COVID-19 is an ongoing challenge, the study results should guide the government in implementing medium- and long-term policy strategies to mitigate the prevalence of psychological distress on the general population. Governments must adopt an extensive crisis prevention and intervention system to improve psychological services and support vulnerable groups, especially women, the young population, and healthcare workers. Without identifying the vulnerable population, the government may not maximize on the development and implementation of appropriate mental and psychological interventions.

## Supporting information

S1 Data (XLSX)
